# Does investment in COVID-19 information systems strengthen national digital health architecture? Lessons learned from Burkina Faso, Indonesia, Mali and Suriname

**DOI:** 10.1093/oodh/oqae001

**Published:** 2024-05-06

**Authors:** Colleen Boyle, Caitlin Madevu-Matson, Agus Rachmanto, Derek Kunaka, Lauren Gilliss, Leah McManus, Madina Kouyate, Maria Aruan, Nony Parmawaty, Rahim Kebe, Vijay Sewradj, Stephanie Watson-Grant

**Affiliations:** Country Health Information Systems and Data Use Project, JSI Research & Training Institute, Inc., 2733 Crystal Drive, Arlington, Virginia, 22202, USA; Country Health Information Systems and Data Use Project, JSI Research & Training Institute, Inc., 2733 Crystal Drive, Arlington, Virginia, 22202, USA; Digital Transformation Office, Ministry of Health of the Republic of Indonesia, Jl. HR. Rasuna Said Blok X5 Kav. 4-9Jakarta Selatan, Jakarta, 12950, Indonesia; Country Health Information Systems and Data Use Project, JSI Research & Training Institute, Inc., 286 Auriga Street, Waterkloof, Pretoria, 0181, South Africa; Country Health Information Systems and Data Use Project, JSI Research & Training Institute, Inc., 2733 Crystal Drive, Arlington, Virginia, 22202, USA; Country Health Information Systems and Data Use Project, JSI Research & Training Institute, Inc., 30th floor, Sampoerna Strategic Square, South Tower Jl., Jend. Sudirman Kav. 45-46, Jakarta, 12930, Indonesia; Country Health Information Systems and Data Use Project, JSI Research & Training Institute, Inc., Mali Quartier Hamdallaye ACI 2000, Rue 317, Porte 140, Immeuble SYLLA, Bamako, Mali; Country Health Information Systems and Data Use Project, JSI Research & Training Institute, Inc., 30th floor, Sampoerna Strategic Square, South Tower Jl., Jend. Sudirman Kav. 45-46, Jakarta, 12930, Indonesia; Country Health Information Systems and Data Use Project, JSI Research & Training Institute, Inc., 30th floor, Sampoerna Strategic Square, South Tower Jl., Jend. Sudirman Kav. 45-46, Jakarta, 12930, Indonesia; Country Health Information Systems and Data Use Project, JSI Research & Training Institute, Inc., Ouaga 2000, Arrondissement 12, Ouagadougou, Burkina Faso; Country Health Information Systems and Data Use Project, JSI Research & Training Institute, Inc., Paramaribo, Suriname; Country Health Information Systems and Data Use Project, JSI Research & Training Institute, Inc., 2733 Crystal Drive, Arlington, Virginia, 22202, USA

**Keywords:** COVID-19, digital health architecture, health information systems, interoperability, vaccination, pandemic response

## Abstract

There is limited research on how emergency investments can support national health information system strengthening both for a given health emergency response and for routine health program areas. This study examined the effect of COVID-19 vaccine digital investments on advancing national digital health system architectures in Burkina Faso, Indonesia, Mali and Suriname. Primary qualitative data were collected through purposively sampled key informant interviews and focus group discussions with 67 key stakeholders from government institutions, donors and implementing partners from February to May 2023. Primary data were triangulated with secondary data collected through desk review and were analyzed deductively and inductively using thematic coding. The study found that although USAID’s COVID-19 vaccine digital investments in Burkina Faso, Indonesia, Mali and Suriname did not contribute to the development of formal national digital health architecture documentation, progress was made in strengthening components of national digital health architectures, specifically around standards and interoperability and around governance. Progress was also made in motivating government stakeholders to invest in formal digital health transformation efforts. Lessons learned from this study suggest that connecting investments in system development and enhancement with investments in governance and infrastructure can improve country data management processes and data use for decision-making in routine health and emergency responses.

Abrégé

Peu de recherches ont été effectuées sur la façon dont les investissements fournis dans les situations d’urgence peuvent appuyer le renforcement du système national d’information sur la santé, tant pour une intervention d’urgence sanitaire donnée que pour les secteurs de programmes de santé courants. Cette étude examine l’effet des investissements numériques dans le vaccin contre la COVID-19 sur le développement des architectures nationales des systèmes de santé numériques au Burkina Faso, en Indonésie, au Mali et au Suriname. Les données qualitatives primaires ont été recueillies au moyen d’entretiens auprès d’informateurs clés échantillonnés de manière ciblée et de discussions de groupes avec 67 parties prenantes clés issues d’organismes gouvernementaux, de donateurs et de partenaires de mise en œuvre entre les mois de Février et Mai 2023. Les données primaires ont été triangulées avec les données secondaires recueillies par le biais d’une étude documentaire et ont été analysées de manière déductive et inductive à l’aide d’un codage thématique. L’étude a révélé que, bien que les investissements numériques de l’USAID en faveur du vaccin contre la COVID-19 réalisés au Burkina Faso, en Indonésie, au Mali et au Suriname n’aient pas contribué au développement d’une documentation officielle sur l’architecture numérique de la santé, des progrès ont été réalisés dans le renforcement de certains éléments des architectures numériques nationales de la santé, en particulier autour des normes et de l’interopérabilité et autour de la gouvernance. Des progrès ont également été réalisés s’agissant d’inciter les acteurs gouvernementaux à investir dans les efforts formels de transformation de la santé numérique. Les enseignements tirés de cette étude montrent que lier les investissements dans le développement et l’amélioration des systèmes aux investissements dans la gouvernance et les infrastructures permet d’améliorer les processus de gestion des données nationales et l’utilisation des données pour la prise de décisions dans les interventions sanitaires d’urgence et de routine.

Resumen

Son limitadas las investigaciones sobre la manera en que las inversiones de emergencia pueden apoyar el fortalecimiento de los sistemas nacionales de información de salud, tanto para la respuesta a una emergencia sanitaria determinada como para áreas de programas de salud de rutina. En este estudio se examinó el efecto de las inversiones digitales en vacunas contra la COVID-19 sobre el avance de las estructuras de los sistemas nacionales de salud digital en Burkina Faso, Indonesia, Malí y Surinam. Se recopilaron datos cualitativos primarios a través de entrevistas con informantes clave muestreadas intencionalmente y discusiones de grupos focales con 67 interesados clave de instituciones gubernamentales, donantes y socios implementadores de Febrero a Mayo de 2023. Los datos primarios se triangularon con datos secundarios recopilados mediante revisión documental y se analizaron de forma deductiva e inductiva mediante codificación temática. En el estudio se halló que, aunque las inversiones digitales de USAID en vacunas contra la COVID-19 en Burkina Faso, Indonesia, Malí y Surinam no contribuyeron al desarrollo de la documentación formal de las estructuras nacionales de salud digital, sí ayudaron a fortalecer ciertos componentes de esas estructuras, específicamente en relación con las normas y la interoperabilidad y con la gobernanza. También se avanzó en la motivación de las partes interesadas gubernamentales para que inviertan en esfuerzos formales de transformación de la salud digital. Las lecciones aprendidas a partir de este estudio sugieren que conectar las inversiones en el desarrollo y la mejora del sistema con las inversiones en gobernanza e infraestructura puede mejorar los procesos de gestión de datos nacionales y el uso de datos para la toma de decisiones en las respuestas de rutina de salud y emergencia.

## INTRODUCTION

A country’s national digital health architecture represents a blueprint or roadmap of data, systems and technologies. When the architecture is planned and documented, it guides governments and their partners to identify health information needs, the technologies and tools that can provide the necessary data and the way in which these pieces fit together to form a comprehensive and coordinated system [1]. This architecture also provides standards and guidelines to identify country-specific technology and governance requirements that can enable interoperability between systems and integration of digital technologies using shared services. It can support routine health service provision and emergency health responses. National digital health architectures can: lower the financial and management burden of competing digital systems through the use of reusable systems or assets; strengthen national health institutions and the provision of health care overall; and promote the effectiveness, reach and cost efficiency of digital investments [2]. When digital solutions are not managed as part of a national digital health architecture, health data are likely to be fragmented, which affects the quality of service delivery and decision-making [3].

There is limited research on how emergency investments can support national health information system (HIS) strengthening both for a given health emergency response and for routine health programs. This study examined the effect of COVID-19 vaccine digital investments on advancing national digital health system architectures in Burkina Faso, Indonesia, Mali and Suriname through qualitative key informant interviews (KIIs) and focus group discussions (FGDs) with key stakeholders from government institutions, donors and implementing partners. The purpose was to provide insight into whether and how technical and financial investments in COVID-19 information systems change a country’s overall digital health enabling environment, establishing whether or not digital investments originally directed to a health emergency can also improve routine HISs and build resilience in health systems against future pandemics.

### Background

Beginning in 2021, the United States Agency for International Development (USAID) Country Health Information Systems and Data Use (CHISU) program began supporting several countries—including Burkina Faso, Indonesia, Mali and Suriname—to improve their COVID-19 data management and use. In these countries, COVID-19 vaccination data capture was managed by national government bodies that operated various data systems in parallel to the health management information system (HMIS) in that country. The subsequent paragraphs describe contextual information on digital health architecture and COVID-19 digital solutions in each of the study countries, primarily gathered through CHISU’s implementation experience.

#### Burkina Faso

CHISU supported strengthening the collection, management and use of COVID-19 data from April 2022 through December 2023 in Burkina Faso. Activities included revising and digitizing data collection tools to include data elements that were needed to quickly respond to the COVID-19 pandemic and managing the administration of the COVID-19 database. CHISU also developed solutions such as dashboards and other data visualization tools in order to facilitate data-based decision-making. Lastly, CHISU contributed to COVID-19 data exchange and integration of data management systems.

Burkina Faso’s HIS includes a variety of data management applications. In 2018, a systems map across health program areas counted more than 81 applications [4]. These software applications are fragmented; they generally collect the same information in different formats and are not interoperable. While there are some national strategic frameworks (including a routine HIS strategic plan and a One Health strategic plan) that provide system and data guidelines, there is no formal national digital health architecture documentation in Burkina Faso.

Burkina Faso’s national routine HMIS or national health data warehouse, Entrepôt National des Données de la Santé (ENDOS), houses all national health program data in District Health Information Software 2 (DHIS2), an open-source, web-based HMIS platform. The One Health information system, also in DHIS2, enables disease surveillance data exchange of zoonotic and human health threats (such as rabies and COVID-19) reported at the community level with ENDOS to support the country’s Ministry of Health (MOH) in delivering a comprehensive and effective response. One Health refers to an approach that addresses health threats at the animal–human–environment nexus [5]. The One Health information system has four instances (each installation of DHIS2 on a server is called an instance), one of which is the MS-Surveillance epidemiological surveillance database managed by Burkina Faso’s MOH. With USAID funding (beginning with the MEASURE Evaluation program prior to the pandemic), the technical secretariat of One Health developed an interoperability layer to connect One Health instances across the ministries of health, environment, and animal resources. This interoperability layer now acts as an enabler of data exchange between multiple One Health instances, including MS-Surveillance and those for environment and animal resources; ENDOS; the community data management application, mHealth; and the mobile application for COVID-19 test results and vaccination cards, COVID-Info.

In response to the COVID-19 pandemic, MS-Surveillance was immediately updated to include COVID-19 data management for case and contact tracing, testing and deaths. Initial paper-based and Microsoft Excel-based tools for collecting COVID-19 vaccination data were rapidly developed as soon as the first vaccines were received. MS-Surveillance was further developed to include laboratory and vaccination data monitoring, appointment booking and payment for PCR (polymerase chain reaction) tests via mobile applications. In January 2023, CHISU mapped data management systems in Burkina Faso and identified 15 total applications being used for the COVID-19 response. Other donors that contributed to COVID-19 digital investments included the World Health Organization; the United Nations Children’s Fund; Gavi, the Vaccine Alliance; the World Bank; the Global Fund to Fight AIDS, Tuberculosis and Malaria; and Agence Française de Développement.

#### Indonesia

CHISU supported strengthening governance for COVID-19 information systems, improving availability and interoperability of quality COVID-19 data, and improving data analysis and use to catalyze improvements in COVID-19 vaccination from August 2022 to November 2023 in Indonesia.

Prior to the COVID-19 pandemic, many data systems existed but used non-standardized variables and metadata (the structure of the data). To support the acceleration of the COVID-19 response at the beginning of the pandemic, Indonesia’s MOH redirected health sector funding to develop COVID-19-specific digital tools, which resulted in myriad data systems for testing, case management and treatment, contact tracing, vaccination and stock management. COVID-19 vaccination data is primarily captured in Pcare COVID (the social health insurance agency’s COVID-19 information system), while COVID-19 case management data is captured in the New All Record (NAR) application. Contact tracing and vaccination certification for border verification and precondition to enter public spaces were captured in the PeduliLindungi app (now SATUSEHAT mobile). In addition to developing these applications, public and private dashboards for the MOH, provincial health offices and district health offices were created to track vaccination coverage progress. The MOH also initiated efforts to update these systems to use national identification numbers as unique identifiers to share individual data on testing, tracing, treatment and vaccination between the various systems.

The lessons learned from the development and use of these systems during the COVID-19 pandemic inspired the Indonesian government to develop the Blueprint for Health Digital Transformation Strategy 2024, which outlines a roadmap for health care technology transformation in Indonesia. This involves standardizing all health data, integrating and interoperating digital health applications and supporting long-term improvement of the digital health system ecosystem for non-emergency health areas and future health emergencies.

At the center of the blueprint is the SATUSEHAT platform—Indonesia’s national platform for health information exchange and data analytics [6]. This platform, launched in July 2022, allows health facilities to exchange data between each other, with other levels of the health system and between health programs. Based on a rapid assessment of COVID-19 information systems, Indonesia’s MOH developed data standards for both COVID-19 case management and vaccination data and plans to use these standards to link COVID-19 data systems with the SATUSEHAT platform. In February 2023, the MOH rebranded the PeduliLindungi app as SATUSEHAT mobile, and expanded its functionality to allow the more than 105 million app users to access their personal health record from their health care providers.

#### Mali

CHISU supported strengthening COVID-19 data quality and use at all levels of the health system from January 2022 through August 2023 in Mali. Activities included improving the coordination of the national crisis committee for COVID-19, which eventually expanded into a health emergency committee; establishing HIS governance structures; expanding the use of the HMIS (DHIS2) for COVID-19 reporting; and improving data quality processes and analytics and use of data to respond to the COVID-19 pandemic.

In Mali, health facilities were quickly overwhelmed by the large amount of individual data produced between 2020 and 2022 due to the COVID-19 pandemic. To address this, Mali’s digital health strategic plan for 2021–25 includes some guidance and recommendations for building a more integrated HIS. However, a rapid assessment conducted by CHISU in March 2022 identified the shortcomings of the data systems set up in response to the COVID-19 pandemic. These included human resources challenges, missing data elements, unaligned data collection tools and challenges contributing to untimely or incomplete data entry in the COVID-19 DHIS2 instance, including internet connectivity issues and data synchronization problems. Other challenges were limited supervision and data quality review, as well as insufficient partner coordination for HIS strengthening. In addition, an interoperability maturity assessment completed by CHISU in 2022 found an absence of national enterprise architecture and interoperability guidance. CHISU supported the creation of an inventory of HIS digital applications and also the development of interoperability guidance.

Initial capture of COVID-19 vaccination data in Mali used Excel sheets; forms were customized in the national DHIS2 system later in 2021. Challenges with server capacity led the country to separate health data onto three servers: one for routine aggregate data (national HMIS), one for surveillance and one for COVID-19. The COVID-19 DHIS2 instance contains DHIS2 Trackers for individual-level data on case-based surveillance, contact tracing, testing and adverse effects reporting and vaccination, as well as aggregate COVID-19 data. As of April 2023, Excel sheets were still used in parallel with DHIS2 for COVID-19 vaccination aggregate data.

While surveillance and vaccination data from the same patients were initially not linked in the COVID-19 DHIS2 instance, CHISU supported configuration of a functionality to link the forms within the system—allowing each client to be tracked across COVID-19 testing, case notification and vaccination. However, no interoperability exists between the national HMIS and COVID-19 DHIS2 instance, and COVID-19 data are still not integrated with routine health data in the national HMIS. Interoperability does exist between the COVID-19 testing Tracker and YNIETTE (an application for COVID-19 testing for travelers), as well as between OSP-Santé (a pharmaceutical management application) and the national HMIS, which excludes COVID-19 data. Other donors that contributed to COVID-19 digital investments included the World Health Organization, the United Nations Children’s Fund, the Global Fund, the World Bank and Muso.

#### Suriname

CHISU supported the improvement of data collection, aggregation, analysis and use of COVID-19 vaccination data from April 2022 to September 2024 in Suriname. Activities included identifying COVID-19 data gaps that hindered timely and effective response to the COVID-19 pandemic and applying appropriate solutions to address issues in the collection, storage and utilization of COVID-19 vaccination data.

Since February 2019, Suriname’s MOH has worked with the Information Systems for Health (IS4H) project, funded through a loan from the Inter-American Development Bank and supported by the Pan American Health Organization (PAHO), to plan for a national health information exchange platform. Prior to the initiation of the IS4H project, there was limited to no interoperability between existing information systems in any health program area. The IS4H project implemented the IS4H Maturity Assessment Tool to assess organizational capacity related to governance, data management, digital transformation, innovation and knowledge management, and produced a roadmap of activities and products to improve capabilities in the aforementioned areas [7]. The country’s MOH continues to work with its national and international stakeholders and donors to mobilize the required resources to address all the products and activities in the roadmap, and to ensure that investments are aligned with PAHO’s Plan of Action for Strengthening Information Systems for Health 2019–2023 for its member states [8].

Prior to the COVID-19 pandemic, health program data were not digitized beyond the use of Excel, requiring manual data entry and management. Initial capture of COVID-19 vaccination data occurred in numerous Excel spreadsheets that were aggregated into a Google Sheet serving as the master list at the central government level. The Surinamese government partnered with the private sector to create an information system to register COVID-19 test results called the COVID database. Although there were different initiatives to digitize the COVID-19 vaccine registry, in May 2021 the MOH selected DHIS2 (also used by the country’s malaria program and supported by PAHO). In July 2021, the vaccine registry was rolled out and has been used to register COVID-19 vaccinations since.

## MATERIALS AND METHODS

### Study design

We designed a qualitative study to examine USAID’s COVID-19 investments’ effect on national digital health architectures in select countries based on CHISU’s participation in a learning group (referred to as the COVID-19 Vaccine Digital Collaborative Learning Agenda, or ‘Learning Agenda’) with three other global USAID-funded projects. The Learning Agenda’s objective was to collectively examine how USAID’s COVID-19 data and digital investments may or may not have changed the digital health enabling environment in a selection of project implementation countries. Of the four learning questions collaboratively developed by the Learning Agenda, CHISU’s implementation activities aligned best with two, one of which served as the guiding question for this study:

To what extent and how have USAID’s COVID-19 health system, data and digital health investments advanced the country’s digital health architecture by lowering the financial and management burden of competing digital systems and by improving interoperability between national digital health systems?

We developed four specific research questions related to the guiding question to examine specific changes to national digital health architectures in study countries, which included:

1. How did investments in national COVID-19 vaccination data systems change the overall interoperability of national digital health systems?
2. How did the level of integration of COVID-19 vaccination data flows change over time?
3. What national digital health architecture lessons were learned from implementing COVID-19 vaccination data systems?
4. To what extent were COVID-19 vaccination data used in decision-making for the COVID-19 response?

#### Analytical framework

The Learning Agenda’s theory of change served as an analytical framework that informed research questions and the design of the KII and FGD guides. The theory of change mapped the relationship between USAID’s COVID-19 vaccination digital investments and potential outcomes related to strengthening the digital health enabling environment and, eventually, the health system [2, Figure 1]. Our research interrogates the pathways between three interventions (implement assessments, develop guidance materials and strengthen architecture and integration) to the immediate outcomes of improved and digitized data collection processes and strengthened architecture and interoperability. We also hoped to demonstrate a pathway to the next highest level of outcomes of supporting COVID-19 vaccination goals and improved use of data and strengthening World Health Organization and the International Telecommunication Union (WHO-ITU) eHealth Building Blocks, namely standards and interoperability. We utilized the theory of change under the assumption that we would not substantiate pathways from interventions to outcomes that fell outside the scope of CHISU implementation activities, and that it would not be feasible within the time frame of the learning activity to substantiate pathways to long-term health system outcomes and impact [2].

We used the HIS Stages of Continuous Improvement measurement framework, the Global Goods Maturity Model and the HIS Interoperability Maturity Toolkit to inform the development of KII and FGD guides to elicit information on areas relevant to digital health architecture in each country [9–11].

#### Study setting and sampling criteria

The study was implemented between January and June 2023, with data collection carried out between February and May 2023. The research team comprised CHISU monitoring, evaluation and learning (MEL) staff based in the United States and CHISU resident advisors from each of the four study countries, who represented a mix of backgrounds and skills relevant to digital health and qualitative research. The study sample included four countries where CHISU received COVID-19 funding to provide support related to digital health architecture, and where the study team was able to obtain required approvals and conduct data collection within the given time frame.

Within those countries, the research team used purposive sampling to identify specific individuals from relevant government units, donors and implementing partners to participate in KIIs and/or FGDs if they met the following eligibility criteria:

(1) Managed and/or led COVID-19-related HIS activities at the national or subnational level, with significant involvement in COVID-19 data management and/or vaccination campaigns
(2) Worked for a technical and/or financial partner who funded or provided technical assistance to the national COVID-19 response
(3) Were at least 18 years of age

### Data collection and analysis

#### Data collection

We collected primary qualitative data through KIIs and FGDs. In general, we prioritized holding KIIs to explore perspectives from one to four participants from the same organization at the same time. However, the majority of KIIs were with one participant. In one study country (Indonesia), we utilized FGDs to maximize time and resources and understand a variety of perspectives from different stakeholders in a group setting. We developed qualitative interview guides for KIIs and FGDs based on our research questions and analytical frameworks. We also collected and used desk review materials to inform the design of the KII and FGD guides. The guides were adapted during implementation based on emerging insights. Desk review materials included available country-specific digital health architecture documents, such as national HIS or digital strategies and technical documentation, as well as internal CHISU work plans, relevant deliverables and reports and communications with CHISU resident advisors and country program staff. The guides were prepared in English by CHISU, validated by all four CHISU resident advisors to ensure relevance to each country’s context, and translated into French and Indonesian.

The research team primarily collected data in person, with a few participants joining virtually over Zoom to accommodate participant schedules. For each KII and FGD, one to two interviewers and notetakers were assigned (one notetaker for KIIs and two to three notetakers for FGDs since each had more than 10 participants). KIIs and FGDs were conducted in French in Burkina Faso and Mali; in Indonesian in Indonesia; and in English in Suriname. Each KII lasted 20 minutes to 2 hours; the two FGDs each lasted ~2.5 hours.

#### Data analysis

Each KII and FGD was audio-recorded and transcribed verbatim in the original language (by the research team when in English, and by professional transcription services when in French and Indonesian). The transcripts were then translated from French to English using DeepL software and staff review, and from Indonesian to English using professional translation services. The research teams took notes during KIIs and FGDs and wrote debrief notes after each KII and FGD. Teams met daily, either online or in person, when KIIs and FGDs were carried out.

We analyzed the data using Dedoose, a qualitative analysis computer software package. Identifying information for KII and FGD participants was removed from the transcripts prior to analysis. The research team developed a codebook of descriptive codes that summarized the main topic of each coded section of the transcript. The descriptive codes were generated using the four primary topics of the KII and FGD guides. Eight main codes (parent codes) and 16 subcodes (child codes) were created in Dedoose for descriptive coding. Descriptive codes were created when a new topic not previously included in the codebook was identified in the data. When coding was completed, descriptive codes were tabulated to identify patterns in the data and relationships among different codes. We also triangulated the primary data with information gathered from relevant desk review materials.

## ETHICS AND CONSENT

The study protocol was granted exemption by the John Snow, Inc. Institutional Review Board (*IRB #23-03E*). Consent to participate was obtained by researchers trained in ethical procedures and was obtained prior to participation in a KII or FGD. Team members shared the consent form electronically with participants ahead of time and read the consent form to participants in English, French or Indonesian; team members also provided time for participants to ask questions and clarify concerns. The team specifically asked if each KII or FGD could be recorded and explained the research team’s procedures for removing identifying information from the resulting written KII or FGD transcripts. The team obtained verbal consent after answering participants’ questions and before beginning the KII or FGD. As described in the consent form provided to the participants, all participants could request to withdraw from participation at any time. All invited participants consented to participate and to have the KII or FGD recorded.

## RESULTS

We conducted 30 KIIs and two FGDs, speaking with 67 individuals across the four selected countries: 15 participants across 12 KIIs in Burkina Faso (one KII had four participants from the same institution); 31 participants across two KIIs and two FGDs in Indonesia (the two KIIs had two participants and one participant, respectively, while the two FGDs had 12 participants and 16 participants, respectively); 12 participants across 10 KIIs in Mali (two KIIs each had two participants); and nine participants across six KIIs in Suriname (one KII had two participants and one had three participants).

The section below synthesizes findings from the KIIs and FGDs across the four countries, according to four emergent themes based on the thematic codes derived from the topics interrogated by our research questions. Table 1 summarizes key information from the four study countries linked to three of the four research questions: the extent of interoperability between COVID-19 and other health program data systems (question 1), the level of integration of COVID-19 vaccination data (question 2) and the existence of national enterprise architecture documentation (question 3). Additionally, it’s important to note that all four study countries had a national digital health and/or an HIS strategy in development or already published. The information we learned related to the third research question on data use for decision-making is not included in Table 1, as the details were more nuanced than what could be captured in the table; this information is elaborated on in the results.

**Table 1:** Summary of key information tied to three research questions from four study countries

Research question theme	Burkina Faso	Indonesia	Mali	Suriname
(1) Interoperability between COVID-19 and other health program data systems	Yes, supported by USAID COVID-19 and other funds (Global Health Security Agenda and Maternal and Child Health/Family Planning).	Yes, supported by others initially with USAID COVID-19-funded support starting in November 2022.	Yes, USAID COVID-19 funds supported the HIS Interoperability Maturity Toolkit assessment and interoperability between YNIETTE and DHIS2.	No.
(2) Integration of COVID-19 vaccination data	Yes, from early on in MS-Surveillance with other One Health data.	Initially no; subsequently, yes, with the national citizenship database (for national identification), telemedicine services and vaccine certificate services.	Initially no; subsequently with other COVID-19 data (can trace unique clients), but not with data for other health areas.	No; standalone system for COVID-19 vaccination.
(3) Existence of national enterprise architecture documentation	No formal document. National consensus on DHIS2 as a platform for One Health and HMIS.	No formal document. Blueprint for Digital Health Transformation Strategy is a high-level vision for architecture and data standards but is not an operational document. Multiple platforms and apps.	No formal document. National consensus on DHIS2 as a platform for HMIS. Excel use persists.	No formal document but planning was underway at the time of this study. Little in digital format.

### Theme 1: Interoperability activities to enable COVID-19 data exchange advanced planning efforts for national digital health architectures

Across all study countries, we learned about a number of interoperability-related efforts—with a primary purpose of enabling COVID-19 data exchange—that participants felt advanced national digital health architecture development. Many of these efforts were initiated in response to the COVID-19 pandemic (including activities with USAID COVID-19 investment and those without it). With non-COVID-19 USAID funding, Indonesia developed the interoperability platform SATUSEHAT and other necessary components—including a terminology service, health worker registry, master facility index and interoperability data standards—that enable national data exchange across many health programs, demonstrating progress toward a national digital health architecture that includes but goes beyond COVID-19. A participant from Indonesia said:

‘Before the pandemic, our information system was just okay and nothing special. Now from the lessons we take from the COVID-19 pandemic, we have a very fast system. Then the system is also interoperable between various information systems so that we can get such a comprehensive picture of the information, so it’s not spread across several parts, and we can do the analysis from start to finish.’

Study participants noted that agreement on minimum standards used and establishment of guidelines for all data systems to create a foundation for future automated exchange have been ongoing, both before and during the COVID-19 pandemic. For example, in Suriname the MOH is planning for standardization of adverse event registration in the Event Suspected to be Attributable to Vaccination or Immunization (ESAVI) module in DHIS2 by acquiring licenses for use of a clinically validated international medical terminology dictionary–thesaurus, Medical Dictionary for Regulatory Activities (MedDRA), and medicinal product information dictionary (WHODrug Global) within the ESAVI module. The module was originally only for registering adverse events supposedly attributable to COVID-19 vaccinations, but now will be configured for all vaccinations and will allow for analysis and comparison across databases [12, 13]. A participant from Suriname summarized the relationship between COVID-19 data management and progress toward eventual interoperability of national health system data: ‘Interoperability is something that is always discussed, but it’s not solely functioning for COVID-19 and another system, it is all systems, because we’re promoting the interoperability of the primary care data, COVID data, other kinds of data.’ Establishing interoperability between existing systems will help to create dashboards for the MOH to visualize and analyze critical health data from national key indicators from several key sources. The dashboards will be expanded and improved as additional information systems are implemented and integrated.

Similarly, a variety of standards and interoperability-related activities in Burkina Faso and Mali were described by study participants. System cleanup of metadata was an initial step toward strengthening digital health architecture by adding data elements that were previously missing from the form but needed for decision-making. Cleaning health facility lists was an additional step taken in Mali as was an HIS interoperability maturity assessment, which informed decisions about interoperability and digital health architecture. Concurrently with the data collection for this study, a situational analysis of the data collection and management systems being used for COVID-19 was being conducted in Burkina Faso with the aim of making recommendations for further customizations to existing systems. A participant in Burkina Faso broadly summarized their view of interoperability-related work: ‘This year we have some funding and support for the development of interoperability documents, norms and standards for a health information system in Burkina Faso. When we put this in place, we’ll describe the vision of interoperability for the information system.’

Participants across study countries commented that a significant factor in shifting from the collection of aggregate data to individual-level data was a need for more specific analyses to understand the success of reaching target populations through vaccination campaigns. Availability of individual-level data enabled close tracking of vaccination for vulnerable groups and linkage of information between vaccination and other COVID-19 services being offered to individuals. However, concerns were raised about data security measures being put in place after individual-level data had already been collected. KII and FGD participants across all countries highlighted concerns about protecting individual health data related to both COVID-19 vaccination and primary health care in electronic health records. A participant in Indonesia commented on how data security considerations may differ between emergency responses and more routine provision of services: ‘When the data is still in aggregate, the security team does not really bother to think about how to secure data. However, when a lot of individual data, personal data is exchanged between systems, that’s where, of course, the security team must look at how to secure these data to avoid any misuse.’

### Theme 2: Proliferation and consolidation of systems and data flow were driven by demand for real-time COVID-19 data

Related to our research question on the change in the level of integration of COVID-19 vaccination data flows over time, demand for real-time COVID-19 vaccination data initially resulted in the proliferation of parallel data systems across all study countries according to study participants. This proliferation increased the burden on health care workers and data analysts to deliver real-time data to decision-makers for use. Additionally, the lack of adequate infrastructure supporting COVID-19 vaccination data systems, as well as human resource capacity to accurately develop and use data systems, presented challenges to streamlining and optimizing the data systems that were developed or used.

In three study countries (Burkina Faso, Mali and Suriname), Excel was selected for initial COVID-19 vaccination data capture because, according to one participant in Suriname, ‘Excel is familiar … you would have to train everyone who will enter data … in DHIS2.’ While widespread familiarity with Excel enabled its immediate use for initial COVID-19 vaccination data capture, in some cases it exacerbated data system proliferation that preceded the COVID-19 pandemic by continuing use of those systems even after another more advanced digital system was adopted. While Excel served as a timely system for collecting initial COVID-19 vaccination data, the number of spreadsheets proliferated to meet new and urgent needs. Excel also did not provide automation functionality that delivers individual-level data to decision-makers, which was needed as the pandemic progressed to most effectively deploy resources and vaccinate the population. In Mali, parallel data collection systems persisted as the pandemic carried on. A participant in Mali explained a case for parallel systems:

‘We're not going to try to overload the digital system, i.e. the current management platform, the current [DHIS2] instance. There has to be a system, a separate COVID instance … Because with all this demand for data and information, it [a separate COVID instance] enabled the players to be on the lookout and try to understand the architecture and know what was missing to try and work with all the COVID data.’

COVID-19 vaccination data systems in Burkina Faso and Indonesia were quickly digitized into distinct systems for case management, vaccinations, and logistics management in DHIS2 (Burkina Faso) and across a number of other applications (Indonesia). However, later in the pandemic some of USAID’s COVID-19 vaccine digital investments supported consolidation of data systems and streamlining data flows. In Burkina Faso, COVID-19 vaccination data flow was further consolidated through the automated exchange of information with other systems. Similarly, Indonesia is working to assess the interoperability readiness of COVID-19 vaccination information systems and to build data standards that will be included in the SATUSEHAT platform so that these systems will be interoperable in the long term. In Suriname, the MOH adopted a DHIS2 Tracker as the platform for vaccination registry after having cleaned and imported existing vaccination data originally collected in Excel. And in Mali, a participant said, ‘During the revision of the national plan for the development of vaccines, it was recommended to migrate squarely to DHIS2’—indicating that consolidation of parallel systems continues to be an issue that takes time to resolve.

### Theme 3: No formal enterprise architecture documentation exists, but some efforts to advance it were present

Initially gathered through desk review and validated with KII and FGD participants, we confirmed that no formal enterprise architecture documentation existed in any of the study countries at the time of data collection. Rather than directly contributing to formal documentation activities, the COVID-19 vaccine digital investments we discussed with study participants were used to conduct a variety of activities that are initial steps toward informing future decision-making related to digital health architecture in our study countries.

In Burkina Faso and Mali, participants indicated that although there are no efforts directed toward the development of formal architecture documentation, specific DHIS2 instances serve as the informal digital health architecture to which HIS stakeholders direct investments for continued optimization. In Burkina Faso, strengthening efforts are centered on both ENDOS and the One Health system as the guiding architecture elements for HIS strengthening. A participant noted:

‘I would say that yes, there is an enterprise architecture in Burkina Faso, because of course we have our national health information system, which is based on and uses the ENDOS platform … We must ensure interoperability with the national system or the parent platform which is ENDOS. I believe that all the actors have understood this and we are working in this direction.’

Another participant described how sustained efforts on the One Health system have enabled it to become part of the informal architecture: ‘We’ve used this platform, we’ve perpetuated it, and that in fact allows the One Health project to be sustainable, because you’ve seen that [that’s] the platform being used today for One Health.’ In Mali, a participant noted that DHIS2 became the informal architecture because of its widespread use: ‘Well, the national strategy of the health information system is the use of DHIS2 at all levels, from the central level to the community level. Everyone uses DHIS2 as a source for reporting immunization data, whether it is routine or not.’

Coordination among key HIS stakeholders, especially between government representatives and donor partners, was mentioned by participants as an important factor in the national digital health architecture development process. In Indonesia, interview participants said the Digital Transformation Office was established ‘to invest in a team that is more professional and more mature in terms of technology in order to accelerate the development of the system’ and catalyzed collaboration among government, the private sector and donor partners to support digital transformation of the health sector. Similarly in Suriname, decision-making related to system selection for COVID-19 vaccination data was informed by discussions on the development of a national digital architecture roadmap through close coordination between the MOH and PAHO. Study participants identified opportunities for key HIS stakeholders to communicate with each other through technical working groups; participants identified this as an important determinant of progress for digital health architecture development, whether working toward formal architecture documentation or not.

While HIS-strengthening efforts in Indonesia have not been directed toward formal architecture documentation as an output, the government in Suriname decided to pursue it. The country’s MOH closely collaborated with the IS4H project and other donors to implement its Agenda for the Digital Transformation in the Health Sector, established in 2019 and described by a study participant ‘to see how we can move the system from paper-based to transform it digitally so that data is available and the country can use this data for decision-making.’ While the maturity of the digital health environment is quite different between these two countries (from dozens of disparate digital health applications within multiple information systems in Indonesia to an emergent digital health ecosystem in Suriname), in both cases efforts to advance national digital health architectures prior to and following the crisis response period of COVID-19 were set in motion.

### Theme 4: Persistent data quality concerns did not prevent data use in the COVID-19 response, but directed stakeholders to choose certain data sources for their decision-making

Participants in two study countries (Burkina Faso and Mali) raised concerns related to data quality when asked how COVID-19 vaccination data were used in decision-making for the COVID-19 response. Their concerns were primarily focused on timeliness and completeness. In Burkina Faso and Mali, a lack of sustained internet connectivity was noted as a contributing factor to delays in data entry. Participants said that delays in data entry for COVID-19 cases and vaccination contributed to a mismatch between data reported in Excel and those reported in MS-Surveillance in Burkina Faso.

A participant in Burkina Faso also noted how digitizing data collection tools provided an opportunity to reduce the occurrence of some completeness issues:

‘Sometimes we can end up with notification forms that are not completely and well filled out. So if the tool is poorly filled out, when you enter the data into the database, you will have missing variables. Later on, with DHIS2, as these supports were digitized, we set up mandatory entries where sometimes if you don’t fill in the information, you can’t pass. So this forces the agents in the field to fill in the tools properly and it reduces the problems a little bit.’

Additionally, there were missing data elements (such as disaggregation by patient gender, age, occupation and geolocation) in data collection tools that were identified later in the COVID-19 response that would enable more robust data analysis for the response. According to one participant, ‘There were certain needs in terms of reporting that were difficult to satisfy, but that was mainly because, when the forms were being defined, these needs hadn’t been expressed. Some needs came later.’

In Mali, participants also mentioned that issues with inconsistent or incomplete training contributed to data quality issues. A participant described inconsistencies in how Excel files were used to collect COVID-19 vaccination data due to inadequate training: ‘People did not all use the same [Excel] files … so the compilation becomes difficult. So there was not a standard file used despite the files sent to the field. There were regions that were using other types of files, some were making changes to the files. So that made it difficult to compile at the agency for the central level.’ However, participants also indicated that providing more comprehensive data quality training when training new users in DHIS2 during emergency contexts (like the COVID-19 pandemic) could be used for routine health contexts. One participant in Burkina Faso said, ‘When we train people on DHIS2, there are many DHIS2 applications that will certainly benefit other health programs, that’s for sure! It’s true that we often acquire equipment for COVID, but in reality it can also be used for other health programs, which is why I think that investments made in COVID can also benefit other health programs.’

In spite of data quality challenges, decision-makers moved forward with decision-making related to the COVID-19 response. One participant in Mali shared how data was used for cost-saving decisions about vaccination campaigns:

‘You know, there are many strategies that are used: door-to-door, campaigns, etc. and mass vaccination sites … We realized that mass vaccination sites were very expensive. And in classic campaigns, we had no more data than in mass vaccination sites, even though this strategy cost us less than mass vaccination sites. So, we finally stopped. We did it [mass vaccination] once, but then we said we wouldn’t do it again.’

Another participant shared an example of how stock management data was used in decision-making related to the COVID-19 response both in Mali and a neighboring country. Mali’s MOH realized it ‘could not consume the entire AstraZeneca quantity before it expired. So, we needed to send this to Côte d’Ivoire because Côte d’Ivoire needed it. Analysis allowed for decisions to avoid losses.’

In Indonesia, participants primarily spoke about how the data available in the various COVID-19 data systems and applications were used to optimize COVID-19 vaccination efforts. As one participant shared:

‘We can understand how much is in stock, how much is being used, then we can also monitor how many vaccines are approaching ED [expiry date], and so on. Then, it also helps us to allocate vaccines from one place to another. This means that maybe in one place, there might still be a lot of vaccines, and maybe it’s a bit close to expiration date, thus we can redistribute it to other places that really need it.’

In Suriname, issues with timeliness and completeness were raised by participants when describing use of Excel spreadsheets to collect COVID-19 vaccination data at the beginning of vaccine delivery, but these concerns were lessened with the adoption of DHIS2.

## DISCUSSION

### Stakeholder coordination

The key HIS stakeholders who participated in our study across the four countries provided insight into how COVID-19 vaccine digital investments affected national digital health architecture development efforts beyond what is available in the literature. Productive stakeholder coordination was identified as a key enabling factor for national digital health architecture development. When achieved, coordinated partner support contributed to consolidation and standardization to reinforce integration of data flows and interoperability of systems. Investing in stakeholder coordination will support long-term digital transformation that better prepares countries to respond to future health emergencies.

### COVID-19 data use and governance

All the country examples included in this paper on digital health architecture showed that demand for data drove decisions about data systems and their architecture. The urgent priority in the beginning of the COVID-19 vaccination response was for real-time data for governments to effectively manage vaccination delivery and publicly demonstrate vaccination achievements, as well as report to any donors on the successful use of vaccines in the emergency response. Where there was significant external investment, technical partners and donors also demanded this real-time data for their own accountability. This prioritization of readily available data, and a lack of coordination among institutions managing different parts of the emergency response, led to initial use of parallel, nonintegrated systems in all countries to varying degrees—and a reliance on aggregate data for quick entry and transmission using trusted methods like Excel.

However, over the course of three years, demand for more precise information on outstanding gaps in vaccination coverage—and a recognition of the inefficiency of using multiple disparate systems—led to increased collection and use of individual-level data and greater consolidation of applications and data flows. While COVID-19 data became readily available in real time, data quality issues persisted but did not slow decision-makers from using the data to improve the pandemic response in their respective countries. When a parallel system is established, governance mechanisms are vital to ensure it captures agreed-upon requirements—to avoid proliferation for other data needs—and will be integrated into the wider system and/or retired. Health system stakeholders should consider pairing investments in systems with investments in governance to ensure coordination between the different groups managing and supporting various components and to ensure reinforcement of standards and architecture.

### Digital health architecture

None of the countries in this study had a digital health enterprise architecture in place at the beginning of the COVID-19 pandemic. The absence of formal national architecture documents outlining agreed-upon standards and procedures may explain the development of such a large number of health data management applications that contained duplications and other inefficiencies. Subsequent application mapping assessments by CHISU to identify opportunities for system alignment for COVID-19 in Burkina Faso (2023), Indonesia (2023) and Mali (2022) may not have been needed if national digital health architectures had been in place. It is not surprising that emergency funding, such as USAID’s COVID-19 vaccination support, did not lead to the development of formal enterprise architecture documentation, since routine (non-emergency) settings provide more suitable opportunities for making formal, broadly applicable changes to digital health architecture.

Nonetheless, some of the emergency funding did contribute to landscape analyses, application inventories and other standards to inform future architecture discussions and decisions. Additionally, lessons learned from the COVID-19 response motivated government stakeholders (including the highest levels of government) to invest in formal digital health transformation efforts, which were already underway in Indonesia and Suriname at the time of this study, and to form strong partnerships with the private sector to reach its goals. Governments, donors, implementing partners and the private sector should examine whether their non-emergency investments in the broader digital health enabling environment deliberately feature plans for digital health architecture for routine health contexts as well as for pandemic preparedness.

### Theory of change

Our results provide evidence substantiating the theory of change pathways from three interventions (assessments implemented, guidance materials developed and architecture and integration strengthened) to the immediate outcomes related to data collection, information systems and processes (specifically improved and digitized data collection processes and strengthened architecture and interoperability) and to the intermediate outcomes of supporting COVID-19 vaccination goals and improved use of data—with some initial indications that specific priority populations have been reached. While Indonesia established their Blueprint for Health Digital Transformation Strategy prior to USAID’s COVID-19 vaccination support, we do not see strong evidence yet for progress toward achievement of the broader digital health enabling environment outcomes outside of COVID-19 vaccination as a result of USAID’s COVID-19 investments across any of the study countries. However, current work supported by USAID toward COVID-19 system architecture and governance bodies in all four countries is likely to contribute in the near future to at least one or more of the WHO-ITU eHealth building blocks of leadership and governance, standards and interoperability and services and applications.

### Contextual factors

The study results demonstrate that investments are needed to bolster the contextual factors—such as political will/stability, sustainable funding and connectivity—outlined in the theory of change that support the substantiation of the intervention-to-outcome pathways. For example, improved governance promotes development of and adherence to a robust digital health architecture, especially after initial proliferation of parallel systems during an emergency response to meet immediate needs for decision-making. Sustainable funding for interventions was shown as a strength in Indonesia, where the national budget was used to invest in digital transformation but with complementary support from partners. In Mali and Suriname, extensive external funding was needed. In terms of political stability, both Burkina Faso and Mali saw the formation of new governments that led to changing priorities and staff turnover. In addition, while strengthened eHealth building blocks is an intermediate outcome in the theory of change, participants said that it was also a contextual factor that facilitated other digital health interventions when present, as reliable internet connectivity and working devices (representing the eHealth building block of infrastructure) ensure that COVID-19 vaccination data can be collected in real time. Health system stakeholders should address infrastructure when making investments in HIS strengthening, as multiple participants highlighted that inadequate infrastructure is a barrier to advancing the development of a national digital health architecture.

### Limitations

Our study had some limitations related to design and implementation. Each country’s digital health environment is unique, as is the community of stakeholders involved in strengthening it. While we invited all relevant stakeholders to participate in KIIs and FGDs, the number of participants from each country varied widely, which meant that more qualitative data was produced for some countries compared to others. We tailored and adapted some questions within the KII and FGD guides to best fit participants’ context.

Additionally, while we sought to best synthesize common themes of how USAID’s COVID-19 investments changed the national digital health architecture across the study countries, comparing findings across countries without contextualization should be done with caution. Lastly, we conducted interviews in three languages (English, French and Indonesian) but not Dutch, limiting the opportunity for responses to be given in a primary language of participants in Suriname. Because transcripts were translated into English for analysis, it is possible that some nuance in the meaning of the response in the original language may have been lost.

## CONCLUSION

Although USAID’s COVID-19 vaccine digital investments in Burkina Faso, Indonesia, Mali and Suriname did not contribute to the development of formal documentation of a national digital health architecture, some progress was made in strengthening components of the national digital health architecture. Advancements were demonstrated in the enabling of data exchange between previously disparate COVID-19 data systems, and in planning for future data exchange with other health program data systems. Additionally, demand for real-time COVID-19 vaccination data was identified as a driver of data system proliferation and eventual consolidation, reducing duplication. These contributions demonstrate how COVID-19 vaccine digital investments strengthen the digital health enabling environment, especially around standards and interoperability and around governance. Lessons learned from this study can inform future donor and government investments in HIS strengthening to connect investments in system development and enhancement with governance and infrastructure support—which can then improve country data management processes and data use for decision-making in routine health and emergency responses.

## Data Availability

The data underlying this article will be shared upon reasonable request to the corresponding author.
